# Gene expression analysis of head and neck squamous cell carcinoma survival and recurrence

**DOI:** 10.18632/oncotarget.2772

**Published:** 2014-11-16

**Authors:** Xu Zhi, Katarzyna Lamperska, Paweł Golusinski, Nicholas J. Schork, Lukasz Luczewski, Tomasz Kolenda, Wojciech Golusinski, Michal M. Masternak

**Affiliations:** ^1^ Department of Obstetrics and Gynecology, Center for Reproductive Medicine, Peking University Third Hospital, Beijing, China; ^2^ College of Medicine, Burnett School of Biomedical Sciences, University of Central Florida, Orlando, FL, USA; ^3^ Deptartment of Cancer Genetics, Greater Poland Cancer Centre, Poznan, Poland; ^4^ Department of Enviromental Biology, Poznan University of Medical Sciences, Poznan, Poland; ^5^ Human Biology, The J. Craig Venter Institute, La Jolla, CA, USA; ^6^ Department of Head and Neck Surgery, Greater Poland Cancer Centre, Poznan University of Medical Sciences, Poznan, Poland; ^7^ Postgraduate School of Molecular Medicine, Medical University of Warsaw, Poland

**Keywords:** PCR array, cancer pathway, gene expression, oral cancer

## Abstract

The squamous cell carcinomas represent about 90 % of all head and neck cancers, ranking the sixth most common human cancer. Approximately 450,000 of new cases of head and neck squamous cell carcinoma (HNSCC) are diagnosed every year. Unfortunately, because of diagnosis at the advanced stages and early metastasis to the lymph nodes, the HNSCC is associated with very high death rate. Identification of signature biomarkers and molecularly targeted therapies could provide more effective and specific cancer treatment, prevent recurrence, and increase survival rate. We used paired tumor and adjacent normal tissue samples to screen with RT² Profiler™ PCR Array Human Cancer PathwayFinder^TM^. Total of 20 up-regulated genes and two down-regulated genes were screened out. Out of 22 genes, 12 genes were subsequently validated to be significantly altered in the HNSCC; the samples were from all 41 patients. Five year survival and recurrence selected genes that could represent the biomarkers of survival and recurrence of the disease. We believe that comprehensive understanding of the unique genetic characteristics of HNSCC could provide novel diagnostic biomarkers and meet the requirement for molecular-targeted therapy for the HNSCC.

## INTRODUCTION

Cancer is one of the leading medical causes of death worldwide, and advanced medicines are not promising enough to conquer this devastating illness. Oral cancer or malignant oral mucosa, a tumor that occurs in any part of the mouth, is commonly categorized under the head and neck cancer, ranking the sixth most common human cancer [1]. While approximately 54,000 Americans will be diagnosed with HNSCC in 2014, and over 450,000 new cases are found every year worldwide (http://oralcancerfoundation.org). The disease is particularly associated with high death rate; approximately 13,500 deaths are reported every year in the USA. The disease is diagnosed at very advanced stage in the majority of patients, and this accounts for the very high death rate, approximately 43 % at five years from diagnosis.

Among different type of HNSCC cancers, approximately 90 % cases are of squamous cell carcinomas [2]. HNSCC cancer can be classified according to the anatomical site of the tumor. Metastasis to the local lymph nodes and distant metastasis indicate lower survival rate and poor prognosis. Although tobacco and alcohol are considered as main causes leading to HNSCC [3], there are also other risk factors, including mechanical stimulation, human papillomavirus for the oropharyngeal tumors, and some genetic predisposition, known to be associated with the disease. It was recently shown that HPV infection might be responsible for accommodating TP53 mutations affect the expression of TP53 [4]. The same group identified several changes underlying downregulation of tumor suppressive effect of TGF-β in HNSCC [4]. Early-stage tumors are treated with surgery or radiotherapy and have a favorable prognosis. The mainstays of treatment for advanced tumors are surgery combined with postoperative radiotherapy. In the past decade, the role of organ-preservation protocols, with combined chemoradiation and surgery for salvage has increased. Molecularly targeted therapy is expected to be more effective and more selective towards cancer cells minimizing normal cell damage; however, targeted therapy involves genome-based individualized treatment of patients suffering from cancer. Importantly, the determination of genetic signature and potential biomarkers for the aggressiveness of HNSCC would allow predicting the chances of survival or recurrence of the disease. Developing potential important biomarkers would allow more precise therapeutic approach for each individual case during the treatment. Therefore, in order to understand the genetic regulation of HNSCC, we examined the gene expression levels in HNSCC affected tissues as well as the healthy tissue from the same patients; we correlated our findings with five years survival follow-up.

## RESULTS

### Cancer Pathway Analysis

**Figure 1:**
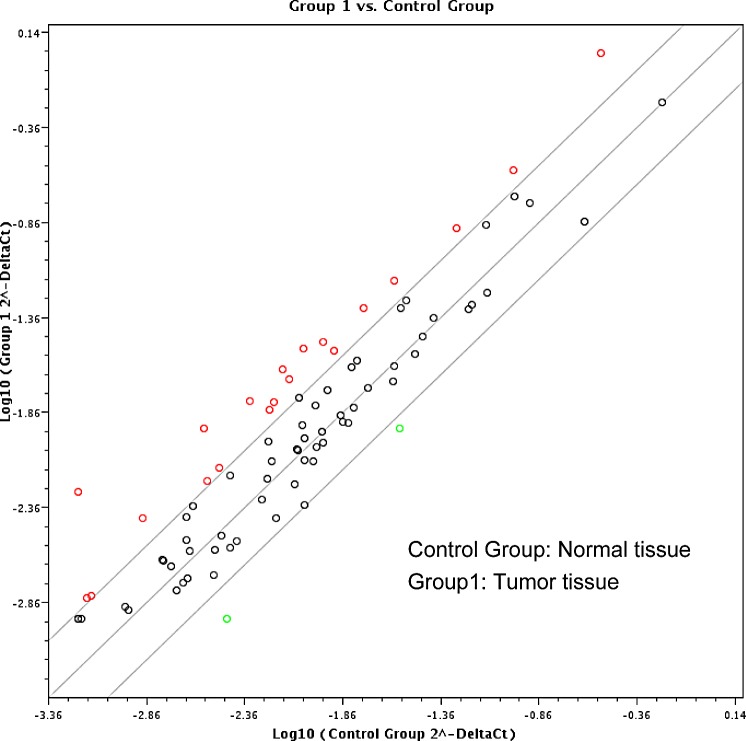
Screening by RT² Profiler™ PCR Array Human Cancer PathwayFinderTM (PAHS-033Z) Eighty-four cancer related genes were analyzed using RT² Profiler™ PCR Array (n = 5 per group). Twenty-two genes were identified with more than two-fold changes between malignant and normal oral mucosa, shown in red and green circles, respectively.

Paired HNSCC and adjacent normal tissue samples of five randomly selected patients were used for the screening with RT² Profiler^TM^ PCR Array Human Cancer PathwayFinder^TM^. Considering a standard two-fold increase or decrease of RNA expression between tumor and healthy tissues to be biologically significant, performed analysis indicated upregulation of 20 analyzed genes including ACLY (ATP citrate lyase), BCL2L11 (BCL2-like 11), CA9 (carbonic anhydrase IX), CDC20 (cell division cycle 20), CDH2 (cadherin 2, type 1, N-cadherin), ETS2 (v-ets avian erythroblastosis virus E26 oncogene homolog 2), FOXC2 (forkhead box C2), IGFBP3 (insulin-like growth factor binding protein 3), KRT14 (keratin 14), LDHA (lactate dehydrogenase A), MCM2 (minichromosome maintenance complex component 2), MKI67 (antigen identified by monoclonal antibody Ki-67), PGF (placental growth factor), PPP1R15A (protein phosphatase 1, regulatory subunit 15A), SERPINB2 (serpin peptidase inhibitor, clade B (ovalbumin), member 2), SKP2 (S-phase kinase-associated protein 2, E3 ubiquitin protein ligase), SLC2A1 (solute carrier family 2, member 1), SNAI2 (snail homolog 2 (Drosophila)), STMN1 (stathmin 1), and VEGFC (vascular endothelial growth factor C), and down-regulation of only two genes including OCLN (occludin) and SOX10 (SRY-box 10)(Fig. 1).

### Validation with individually optimized real-time PCR system

**Figure 2:**
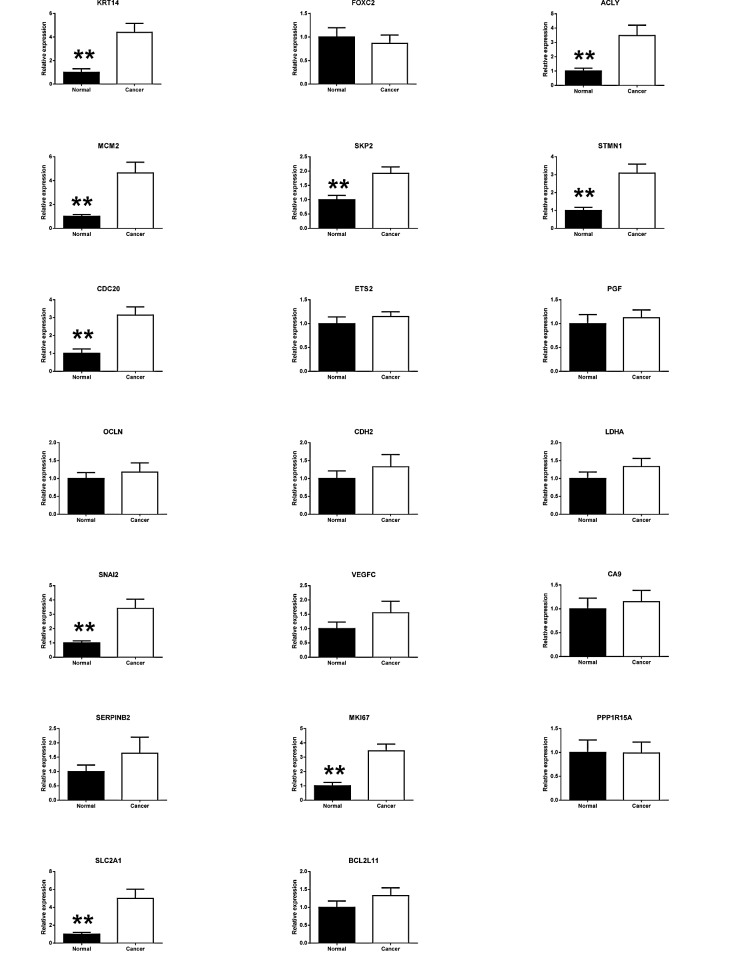
Validation of the PCR screening results Total 22 genes, screened out by RT² Profiler™ PCR Array, were validated using real-time PCR in a larger patient number (n = 41 per group). Ten genes showed significant changes between paired tumor and adjacent normal tissues. SOX10 gene showed extremely low expression and was excluded from further investigation (*p < 0.05; **p < 0.01, *t*-test).

Twenty selected genes that indicated alterations in initial five patients during RT² Profiler™ PCR Array Human Cancer PathwayFinderTM analysis were individually optimized by real-time-PCR (Fig. 2). Regardless of tumor sites, paired t-test analysis of tumor and healthy tissues from all 41 patients indicated significant expression changes in nine genes including ACLY (p = 0.00110), CDC20 (p = 0.00007), KRT14 (p = 0.00031), MCM2 (p = 0.00011), MKI67 (p = 0.00004), SKP2 (p = 0.00012), SLC2A1 (p = 0.00012), SNAI2 (p = 0.000862), and STMN1 (p = 0.00031) (Table 1). IGFBP3 also showed significant difference, as reported previously [5]. At the same time, the real-time PCR analyses for all 41 patients did not show any significant alterations of CA9, CDH2, ETS2, LDHA, PGF, PPP1R15A, SERPINB2, VEGFC, OCLN FOXC2 and BCL211 genes in tumor tissues when compared with healthy tissue samples (Table 1)(Figure 2).

**Table 1:** 

				Survival Analysis of Death						Survival Analysis of Recurrence					
		Normal-Tumor Difference		Normal Tissue		Tumor Tissue		N-T		Normal Tissue		Tumor Tissue		N-T	
Gene #	Gene	N-T Ave	p-value	Coeff	p-value	Coeff	p-value	Coeff	p-value	Coeff	p-value	Coeff	p-value	Coeff	p-value
1	KRT14	−3.40	0.00031	0.25	ns	0.86	ns	−0.68	ns	0.52	ns	−0.19	ns	0.31	ns
2	FOXC2	0.13	0.53660	0.7	ns	−0.89	ns	1.12	ns	−0.99	ns	−1.43	ns	0.87	ns
3	Acly	−2.48	0.00110	−0.45	ns	−1.29	ns	1.22	ns	0.5	ns	0.23	ns	−0.04	ns
4	MCM2	−3.65	0.00011	−0.25	ns	−1.74	0.08	1.72	0.085	0.57	ns	−0.1	ns	0.20	ns
5	SKP2	−0.92	0.00012	0.37	ns	−1.59	ns	2.11	0.034	0.39	ns	−0.73	ns	1.40	ns
6	STMN1	−2.08	0.00031	−0.29	ns	−1.37	ns	1.25	ns	1	ns	0.89	ns	−0.70	ns
7	CDC20	−2.14	0.00007	0.09	ns	−0.84	ns	0.84	ns	1.46	ns	0.87	ns	−0.48	ns
8	ETS2	−0.15	0.33970	−0.21	ns	−2.00	0.046	1.18	ns	0.34	ns	0.12	ns	0.26	ns
9	PGF	−0.12	0.54670	0.7	ns	−0.86	ns	1.33	ns	−0.14	ns	−0.68	ns	0.57	ns
10	OCLN	−0.18	0.42000	0.28	ns	−0.22	ns	0.43	ns	−0.98	ns	−0.7	ns	0.12	ns
11	CDH2	−0.33	0.27150	0.52	ns	−0.39	ns	0.83	ns	−0.7	ns	−0.62	ns	0.32	ns
12	LDHA	−0.33	0.13650	0.51	ns	−0.21	ns	0.62	ns	−0.55	ns	−1.08	ns	0.80	ns
13	SNAI2	−2.14	0.00086	−0.34	ns	−0.62	ns	0.54	ns	0.6	ns	0.47	ns	−0.36	ns
14	VEGFC	−0.55	0.12160	0.29	ns	−0.22	ns	0.46	ns	−0.64	ns	−0.69	ns	0.47	ns
15	SERPINB2	−0.63	0.27060	−0.01	ns	−0.14	ns	0.13	ns	0.64	ns	0.71	ns	−0.45	ns
16	MKI67	−2.44	0.00004	−0.16	ns	−0.64	ns	0.54	ns	0.8	ns	0.35	ns	−0.09	ns
17	PPP1R15A	0.01	0.96620	−0.49	ns	−1.45	ns	0.64	ns	1.03	ns	0.63	ns	1.11	ns
18	SLC2A1	−4.00	0.00012	−0.95	ns	−0.98	ns	0.89	ns	−0.01	ns	0.4	ns	−0.44	ns
19	BCL2L11	−0.33	0.15770	0.45	ns	−1.39	ns	1.86	0.062	−0.29	ns	−1.45	ns	1.53	ns
20	CA9	−0.15	0.56150	0.2	ns	−1.00	ns	0.98	ns	−0.14	ns	−0.95	ns	0.79	ns

### Survival analysis for death and recurrence

The patients were followed up for five years after the surgical treatment, recording both death and survival information [5]. We used CPH models to assess the statistical significance of associations between important covariates such as age and sex of the patients (see Methods). We found evidence for a significantly higher risk of death for patients with tumors located in the larynx (p < 0.01). We also found evidence for an association between the risk of recurrence and tumor location at the larynx as well as age (p = 0.05 and p = 0.002, respectively) (Table S1).

### Univariate analysis of gene expression levels

We also assessed the significance of the association between expression levels of each gene and survival and recurrence using CPH models while accommodating covariates (See Methods). For studying 20 genes, we performed 20 univariate analyses. We found significant difference in expression level between normal and tumor tissues for the *SKP2* gene associated with survival (p = 0.034). We found that the greater the expression level in the tumor relative to the normal tissue the higher the risk of death. *SKP2* was the only gene that resulted in a p-value < 0.05 among all 20 genes (exhibiting this kind of association) we analyzed. Interestingly, the *ETS2* gene exhibited a negative correlation between its expression level in tumor tissue and survival (p = 0.046). Nevertheless, we did not find any evidence for association between any of the genes we analyzed and recurrence of cancer in these univariate analyses (Table 1).

### Multivariate analysis of gene expression levels

We also attempted to examine whether there are associations between all 20 genes' expression levels and survival and recurrence using a single multivariate CPH model analysis that also accommodated covariates (see Methods); this model considers the simultaneous influence of the genes on survival and recurrence. For survival, the difference between normal and tumor gene expression levels was not predictive for any gene. However, the expression levels of many genes in the tumor tissue alone were found to be associated with survival. These genes include KRT14, FOXC2, ACLY, PGF, OCLN, CDH2, LDHA, VEGFC, BCL2L11and CA9, (p = 0.037, p = 0.028, p = 0.037, p = 0.0094, p = 0.013, p = 0.210, p = 0.0044, p = 0.030 and p = 0.020, respectively; Table 2)

**Table 2:** 

A	Survival Analysis with Normal Tissue Gene Expression						Survival Analysis with Tumor Tissue Gene Expression							Survival Analysis with N-T Difference in Gene Expression						
	coef	exp(coef)	se(coef)	z	Pr(>|z|)			coef	exp(coef)	se(coef)	z	I Pr(>|z|)			coef	exp(coef)	exp(coef)	z	Pr(>|z|)	
KRT14	0.13713	1.14704	0.23417	0.586	0.558		KRT14	−5.97E-01	5.51E-01	2.86E-01	−2.087	0.03691	*	KRT14	3.38E-01	1.40E+00	4.47E-01	0.757	0.449	
FOXC2	0.31316	1.36774	1.05878	0.296	0.767		FOXC2	6.02E+00	4.13E+02	2.74E+00	2.198	0.02794	*	FOXC2	−7.47E+00	5.71E-04	8.43E+00	−0.886	0.376	
Acly	−0.3108	0.73286	0.62044	−0.501	0.616		Acly	−2.10E+00	1.23E-01	1.01E+00	−2.084	0.03715	*	Acly	7.93E+00	2.79E+03	6.14E+00	1.292	0.196	
MCM2	2.2041	9.06206	1.65332	1.333	0.182		MCM2	6.51E-01	1.92E+00	4.65E-01	1.4	0.16151		MCM2	3.42E+00	3.05E+01	4.02E+00	0.849	0.396	
SKP2	0.06924	1.07169	1.27852	0.054	0.957		SKP2	3.47E+00	3.20E+01	2.36E+00	1.469	0.14185		SKP2	3.12E+00	2.26E+01	2.33E+00	1.335	0.182	
STMN1	−0.27904	0.75651	1.68992	−0.165	0.869		STMN1	8.52E-01	2.35E+00	7.64E-01	1.115	0.26482		STMN1	7.43E+00	1.69E+03	5.84E+00	1.272	0.204	
CDC2D	−0.14991	0.86079	0.57578	−0.26	0.795		CDC20	−8.28E-01	4.37E-01	7.05E-01	−1.174	0.24034		CDC20	4.88E+00	1.32E+02	4.53E+00	1.077	0.282	
ETS2	−0.21309	0.80809	1.27945	−0.167	0.868		ETS2	5.34E+00	2.08E+02	4.07E+00	1.314	0.18898		ETS2	−1.35E+01	1.41E-06	1.03E+01	−1.311	0.19	
PGF	−0.06858	0.93372	2.29869	−0.03	0.976		PGF	9.19E+00	9.83E+03	3.54E+00	2.598	0.00937	**	PGF	7.14E+00	1.26E+03	5.17E+00	1.381	0.167	
OCLN	−0.71081	0.49124	1.76395	−0.403	0.687		OCLN	1.12E+01	7.11E+04	4.50E+00	2.483	0.01303	*	OCLN	−9.02E+00	1.21E-04	8.22E+00	−1.098	0.272	
CDH2	2.89339	18.05439	3.00184	0.964	0.335		CDH2	−4.53E+00	1.08E-02	1.97E+00	−2.301	0.02139	*	CDH2	−4.64E+00	9.70E-03	7.21E+00	−0.643	0.52	
LDHA	−2.54261	0.07866	2.61213	−0.973	0.33		LDHA	1.67E+01	1.70E+07	5.84E+00	2.851	0.00436	**	LDHA	−1.82E+01	1.22E-08	1.45E+01	−1.257	0.209	
SNAI2	−1.59481	0.20295	1.06151	−1.502	0.133		SNAI2	6.46E-01	1.91E+00	3.62E-01	1.785	0.0743	.	SNAI2	−5.23E+00	5.34E-03	3.91E+00	−1.338	0.181	
VEGFC	−1.1118	0.32897	1.31445	−0.846	0.398		VEGFC	−6.57E+00	1.40E-03	3.03E+00	−2.168	0.03016	*	VEGFC	1.71E+01	2.65E+07	1.65E+01	1.034	0.301	
SERPINB2	0.18894	1.20797	0.46295	0.408	0.683		SERPINB2	1.47E+C0	4.35E+00	7.87E-01	1.867	0.06197	.	SERPINB2	−1.54E+00	2.14E-01	1.70E+00	−0.907	0.364	
MKI67	0.98042	2.66557	1.37067	0.715	0.474		MKI67	−7.60E-01	4.68E+01	4.97E-01	−1.529	0.12622		MKI67	−6.00E+00	2.48E-03	6.28E+00	−0.954	0.34	
PPP1R15A	0.46618	1.5939	0.43666	1.068	0.286		PPP1R15A	−3.05E+00	4.74E-02	3.12E400	−0.977	0.32853		PPP1R15A	−5.02E+00	6.58E-03	4.43E+00	−1.134	0.257	
SLC2A1	−2.31027	0.09923	1.72714	−1.338	0.181		SLC2A1	−2.77E-01	7.58E-01	6.80E-01	−0.407	0.68403		SLC2A1	−3.87E+00	2.09E-02	4.06E+00	−0.953	0.341	
BCL2L11	0.04841	1.0496	1.79856	0.027	0.979		BCL2L11	−1.55E+01	1.83E-07	6.64E+00	−2.336	0.01951	*	BCL2L11	9.99E+00	2.17E+04	1.13E+01	0.884	0.377	
CA9	1.18193	3.26067	1.08993	1.084	0.278		CA9	−1.53E+01	2.27E-07	5.38E+00	−2.843	0.00447	**	CA9	8.81E+00	6.67E+03	7.55E+00	1.167	0.243	
sexf0m1l	−0.72355	0.48503	1.74426	−0.415	0.678		sexf0m1	6.48E+00	6.55E+02	3.41E+00	1.9	0.05744	.	sexf0ml	7.50E+00	1.81E+03	1.22E+01	0.617	0.537	
age	−0.04809	1.04927	0.0842	0.571	0.568		age	3.09E-01	1.36E+00	1.73E-01	1.792	0.7312	.	age	1.55E-01	1.17E+00	2.82E-01	0.549	0.583	
layrinx	−0.30401	0.73786	1.46495	−0.208	0.836		layrinx	−2.08E+00	1.25E+01	1.71E+00	−1.217	0.22351		layrinx	−1.15E+01	1.01E-05	8.27E+00	−1.39	0.164	

B	Recurrence Analysiswith Normal Tissue Gene Expression						Recurrence Analysis with Tumor Tissue Gene Expression							Recurrence Analysis with N-T Difference in Gene Expression						
	coef	exp(coef)	se(coef)	z	Pr(>|2|)			coef	exp(coef)	se(coef)	z	Pr(>|z|)			coef	exp(coef)	se (coef)	z	Pr(>|z|)	
KRT14	2.20E-01	1.25E+00	2.90E-01	0.76	0.44748		KRT14	−7.35E-01	4.79E-01	3.50E-01	−2.098	0.0359	*	KRT14	0.1655496	1.179979	0.154861	1.069	0.28522	
FOXC2	−7.59E+00	5.05E-04	2. 80E+00	−2.714	0.00665	**	FOXC2	6.39E-01	1.89E+00	2.68E+00	0.239	0.8115		FOXC2	−0.402407	0.668709	0.86971	−0.463	0.64359	
Acly	3.58E-01	1.43E+00	5.72E-01	0.626	0.53121		Acly	−4.53E-02	9.56E-01	9.08E-01	−0.05	0.9602		Acly						
MCM2	−4.52E-01	6.36E-01	2.05E+00	−0.22	0.82566		MCM2	−1.27E+00	2.81E-01	7.46E-01	−1.704	0.0883	.	MCM2						
SKP2	−4.10E+00	1.65E-02	2.26E+00	−1.815	0.06958		SKP2	2.81E+00	1.66E+01	2.66E+00	1.056	0.2912		SKP2	1.72097	5.589946	1.298089	1.326	0.18492	
STMN1	−1.19E+00	3.05E-01	3.37E+00	−0.353	0.72409		STMN1	1.72E+00	5.59E+00	1.59E+00	1.081	0.2798		STMN1	0.137648	1.147572	0.630316	0.218	0.82713	
CDC20	3.61E+00	3.68E+01	1.33E+00	2.703	0.00686	**	CDC20	−9.25E-01	3.97E-01	1.16E+00	−0.797	0.4253		CDC20	−0.77891	0.458906	0.687483	−1.133	0.25722	
ETS2	5.22E-01	1.69E+00	1.39E+00	0.376	0.70664		ETS2	4.97E+00	1.44E+02	2.50E+00	1.985	0.0471	*	ETS2	0.394798	1.484085	0.963766	0.41	0.68207	
PGF							PGF	8.36E+00	4.25E+03	4.75E+00	1.757	0.0788	.	PGF	−6.460038	0.001565	3.231576	−1.999	0.0456	*
OCLN	−7.49E+00	5.58E-04	3.79E+00	−1.977	0.04803	*	OCLN	4.24E+00	6.92E+01	2.59E+00	1.634	0.1023		OCLN	−0.950177	0.386673	2.205405	−0.431	0.66658	
CDH2	7.13E+00	1.24E+03	5.28E+00	1.35	0.17694		CDH2	−1.06E+00	3.46E-01	2.48E+00	−0.427	0.6693		CDH2	0.225281	1.252675	0.779593	0.289	0.7726	
LDHA	−1.83E+00	1.63E-01	6.72E+00	−0.272	0.78545		LDHA	1.01E+00	2.74E+00	4.57E+00	0.22	0.8257		LDHA	3.789493	44.23399	2.866791	1.322	0.18622	
SNAI2	−3.95E-01	6.74E-01	9.46E-01	−0.418	0.67609		SNAI2	−1.20E-01	8.87E-01	4.08E-01	−0.295	0.7678		SNAI2	0.586288	1.797305	0.328687	1.784	0.07447	
VEGFC	2.74E+00	1.55E-01	2.42E+00	1.135	0.25646		VEGFC	−1.43E+00	2.40E-01	1.73E+00	−0.824	0.41		VEGFC	−1.325276	0.26573	1.413558	−0.938	0.34848	
SERPINB2	1.41E+00	4.09E+00	6.69E-01	2.106	0.0852	*	SERPINB2	1.28E+00	3.59E+00	8.01E-01	1.598	0.11		SERPINB2	−0.626844	0.534275	0.548636	−1.143	0.25323	
MKI67	−1.05E+00	3.53E-01	1.90E+00	−0.551	0.58175		MKI67	8.86E-01	2.43E+00	7.62E-01	1.163	0.245		MKI67						
PPP1R15A	7.81E-01	2.18E+00	4.60E-01	1.697	0.08962		PPP1R15A	−6.96E-01	4.99E-01	1.35E+00	−0.517	0.605		PPP1R15A	0.410646	1.507792	0.499668	0.822	0.41117	
SLC2A1							SLC2A1	−5.31E-01	5.88E-01	8.18E-01	−0.649	0.5166		SLC2A1	−0.336317	0.714397	0.19036	−1.767	0.07727	
BCL2L11	−8.32E-01	4.35E-01	1.57E+00	−0.529	0.59701		BCL2L11	−9.40E+00	8.29E-05	5.99E+00	−1.568	0.1169		BCL2L11	3.935636	51.19471	2.964494	1.328	0.18431	
CA9							CA9	−5.48E+00	4.17E-03	4.96E+00	−1.105	0.269		CA9	0.530099	1.699101	1.060243	0.5	0.61709	
sexf0ml	−5.15E+00	5.80E-03	2.06E+00	−2.501	0.01239	*	sexf0m1	1.44E+00	4.21E+00	1.55E+00	0.926	0.3545		sexf0m1	−1.098413	0.3334	2.07717	−0.529	0.59694	
age	−6.42E-01	5.27E-01	1.97E-01	−3.264	0.0011	**	age	−1.57E-01	8.54E-01	1.42E-01	−1.107	0.2682		age	−0.451194	0.636867	0.153182	−2.945	0.00322	**
layrinx	−2.18E+00	1.13E-01	1.58E+00	−1.378	0.16832		layrinx	−2.39E+00	9.21E-02	1.79E+00	−1.329	0.1838		layrinx	−1.974012	0.138898	1.931518	−1.022	0.30678	

We also considered the analysis of recurrence of cancer, and we found that the level of gene expression in the normal tissue was associated with recurrence for many genes: FOXC2, CDC20, OCLN and SERPINB2 (p = 0.0067, p = 0.0067, p = 0.048 and p = 0.0352 respectively). Analysis of the expression levels of the genes in tumor tissue indicated significant associations between the KRT14 (p = 0.034) and ETS2 genes (p = 0.0471) and recurrence. However, the analysis of the difference in the mRNA expression levels between normal and tumor tissue indicated that the greater the expression level of placental growth factor (PGF) in tumor relative to normal tissue, the greater is the chance for recurrence (Table 2).

### Gene expression in different tumor locations

#### Larynx

Separate analysis including 25 patients with the tumor located at different parts of the larynx indicated significant increase in10 analyzed genes in the tumor tissues when compared to the corresponding healthy tissues from the same patients (Figure S1) The genes that showed increased expression levels included BCL2L11, (p = 0.0121) (known as important regulator of apoptosis) as well as cell cycle regulatory genes such as CDC20, MCM2 MKI67 SKP2 and STMN1 (p = 0.0011, p = 0.0024, p = 0.0010, p = 0.0003, and p = 0.0057, respectively). In addition, the analysis also revealed increase in KRT14 and SNAI2 (p = 0.001and p = 0.0005, respectively) recognized as genes involved in epithelial-mesenchymal transformation, hypoxia response SLC2A1 gene (p = 0.0007), and ACLY one of the regulator of lipid metabolism (p = 0.0194) (Fig. S1).

#### Oral cavity

The analysis of 10 patients with tumors located in the oral cavity revealed increase in cell cycle regulatory SKP2 (p = 0.0214) and metabolic ACLY gene (p = 0.0403) in the tumor tissues compared to the healthy tissues (Fig. S2).

#### Pharynx

We analyzed five patients with the tumor located at different part of the pharynx and found significant increase in cell cycle regulatory CDC20 and MCM2 genes in the tumor when compared with healthy tissue (p = 0.0467and p = 0.0438, respectively). At the same time, the expression of FOXC2 gene was found to be decreased in tumor tissue compared to the healthy tissues (p = 0.0181) (Fig. S3).

### DISCUSSION

Apart from tobacco and alcohol consumption, viral and bacterial stimulations can also lead to the development of the head and neck squamous cell carcinoma (HNSCC). Genetic markers, which would help in determining the risk of development of the disease or predicting the chances for survival and recurrence of the disease, are yet to be identified. It has been well accepted that identification of signature biomarkers for different types of cancer provides better and more precise diagnosis, stage classification, prediction of metastasis, recurrence potential, prognostic outcome, and most importantly, response to therapy for all affected humans [6]. Importantly some of these markers represents large family of genes that can help predict invasiveness of different kind of cancer [7]. Furthermore, with the development in molecularly targeted therapies, the cancer treatment will undergo a revolutionary change with more specific and more effective individualized therapy for each individual cancer patient [8]. Invasive neoplasm usually develops from precancerous lesions lesions, frequently unable to detect during standard patient examination. Standard management for head and neck cancer, involving surgery followed by irradiation, is supplanted by organ preservation protocols, combining chemotherapy and radiation for advanced cases. Oral cancer is associated with very high death rate of ~43 % at five years from diagnosis, because in the majority of cases, patients come at advanced stages of cancer. Additionally, most of the HNSCC patients are elderly people, limiting the possibilities of aggressive chemotherapy or radiotherapy as treatment. Early and accurate diagnosis of the HNSCC is of critical importance. The current gold standard diagnosis is based on histopathology alone; however, it seems to be highly insufficient. Unfortunately, many other diagnostic attempts to characterize oral cancer through analyzing one or only a few biomarkers have failed [6]. Moreover, published studies often lack follow-up after the treatment to determine the correlation of potential biomarkers with survival or recurrence rate.

In the present study, we used commercially available RT² Profiler™ PCR Array Human Cancer PathwayFinder^TM^ as a new strategy for discovering useful molecular biomarkers with a pathway-based system by comparing the changes in genes expression between tumor and healthy tissues from the same patient. Importantly, in order to correlate our findings with the chances of survival and recurrence of the disease, patients were followed up for five years. Employed cancer pathway assay contained 84 genes, which are involved in all currently focused aspects of cancer process including apoptosis, cell cycle, cell senescence, EMT, hypoxia response, metabolism, angiogenesis, DNA damage/repair, and telomeres/telomerase [9]. The cancer-pathway assay screened out 22 genes with either upregulation or downregulation in cancer tissues compared to the adjacent healthy tissues from the same patient. Except for SOX10, which shows extremely low expression level for PCR detection, 21 initially selected genes were validated using individually optimized real time PCR for all recruited patients. Nine out of these 21 genes did not show any significant changes in expression, while rest of the genes showed significantly differential expression between cancer and adjacent normal tissue in different malignant process such as apoptosis (BCL2L11), cell cycle (CDC20, MCM2, MKI67, SKP2 and STMN1), epithelial-mesenchymal transition (EMT) (FOXC2, KRT14 and SNAI2), hypoxia response (SLC2A1), and metabolism (ACLY). Following our initial screening, individually optimized real-time PCR performed on all 41 patients confirmed increased expression of BCL2L11, an apoptosis regulatory gene in malignancy. We also noticed upregulation of main cell cycle regulatory genes, including CDC20, MCM2, MKI67, SKP2, STMN1, as well as genes responsible for ETM including FOXC2, KRT14 and SNAI2. Interestingly, there was a significant increase of SLC2A1gene associated with hypoxia response and ACLY gene associated with metabolism in the tumor tissues compared to the healthy tissue. Based on these data, it might be concluded that the cell cycle and epithelial-mesenchymal transition (EMT) are the most affected pathways in HNSCC. Additionally other study also indicated overexpression of EMT regulating genes including TWIST1, TWIST2, SNAI1 and SNAI2 in HNSCC when comparing with normal mucosae and the study indicated that overexpression of TWIST2 is associated with poor prognosis for the patients [10]. However, indicating cell cycle and EMT as the major and most affected pathway based on our studycould indicate an overstatement, because our further analysis indicated that the expression of these genes either involved significant alterations or different regulation (in absence of any changes) at different sites including the larynx, the pharynx or the oral cavity. Interestingly, we noticed upregulation of BCL2L11, the known apoptotic pathway gene, in the larynx tumors and downregulation of FOXC2 gene, involved in EMT pathway, in the pharynx tumors only. However, both of these genes did not show any alterations when analyzed with respect to the whole group of patients, suggesting that alterations of these genes in any tumorigenic function can be specific to the site of the tumor. All of these changes might play important role presenting crucial future biomarkers considering the fact that eight of the altered genes, including ACLY, CDC20, MCM2, MKI67, SKP2, SLC2A1, SNAI2, and STMN1, are believed to facilitate the tumor growth and metastasis [11-16], while BCL2L11, KRT14, and FOXC2 are responsible for tumor cells survival and dispense. In addition, it has also been reported that increase in BCL2L11 and KRT14 promote apoptosis and inhibit EMT pathway [17-19], while the decrease in FOXC2 in the pharynx inhibits metastasis [20-23]. The KRT14 is known to be downregulated in the esophageal cancer [24-26]; however, we found it to be upregulated in the HNSCC from our patients. All these data show how difficult it is to draw a conclusion based on the genetic studies performed with humans, because each cohort can be different; moreover, different sites and different causes of the HNSCC makes the search for biomarker more difficult compared to the other types of cancers.

It is important to mention that we did not find any statistically significant difference between men and women for normal tissue samples; however, the present study showed male patients having higher tendency of cancer-related gene expression than women in tumor samples, normalized against adjacent normal tissue samples. This finding is in agreement with a previous report that the HNSCC shows gender difference with male dominancy in development of this type of cancer [1] (Figs. S4 and S5).

However, the most important question that we could ask would be: *What does it mean for the patient prognosis?* Are these changes meaningful and how could we use this information to provide better prognosis or more specific therapies? To answer these questions, we carefully followed the recovery of our patients after treatment. Our analysis on the survival and recurrence of a selected population of the HNSCC patients, followed up for five years [5], revealed that the patients with the tumor located at the larynx are at the highest risk of death. More specifically, our analysis indicates that the difference in the expression level of the SKP2 between normal and tumor tissues is associated with survival. We found that the greater the expression of this gene in tumor tissues relative to normal tissues, the greater is the chance of death. In addition, we also found that the expression level of ETS2 gene in tumor tissues was negatively associated with survival, indicating that the lower the expression of this gene, the higher would be the chance of death.

Our multivariate CPH model analyses revealed a group of genes, including KRT14, FOXC2, ACLY, PGF, OCLN, CDH2, LDHA, VEGFC, BCL2L11and CA9 having expression levels in the tumor tissues, predicting survival. Moreover, we found the expression levels of the genes, FOXC2, CDC20, OCLN, and SERPINB2 in normal tissues was negatively correlated with recurrence; the lower expression of these genes was associated with a higher chance of recurrence. The greater the expression level of the KRT14 and ETS2 genes in the tumor tissues relative to the normal tissues, the greater would be the chance of recurrence. Finally, we also found that the greater the expression level of the PGF gene in the tumor tissues relative to the normal tissues, the greater would be the chance of recurrence of cancer.

In summary, the cancer pathway analyses highlighted several genes showing significant changes in expression in the HNSCC compared to the healthy tissues, suggesting unique molecular pathway involved in the HNSCC. More importantly, the survival and recurrence analyses provided the evidence that some of these genes can be used as important factors for predicting the success of patient recovery or for providing more specific and precise treatments based on molecular analysis. Because of the complicated nature of the HNSCC including environmental stimulants and involvement of different sites, further study should be carried with larger cohort with at least five year of follow-ups to determine not only the differences between tumor and healthy tissues but also to reconfirm and determine new potential biomarkers that would help in designing better therapeutic approaches.

### MATERIALS AND METHODS

#### Patients

Forty-one patients diagnosed with head and neck cancer and subjected to primary surgical treatment in the Department of Head and Neck Surgery, Greater Poland Cancer Center were included in the experiment. All participants were not treated with chemo- or radiotherapy prior to surgical intervention. Among 41 participants, there were 28 men and 13 women with average patient's age of 59 years (ranging from 37 to 70 years). Fresh frozen samples collected during the surgery included cancer tissue; as a control, normal epithelium tissue was also collected within the range of 2 cm distal from the tumor margins from the same patient. The samples were divided into three categories according to the localization of tumor: larynx (n = 25), oral cavity (n = 10), pharynx (n = 5), and other/salivary gland (n = 1). Tumor grading was evaluated following the World Health Organization (WHO) criteria and the TNM (T: size of tumor; N: degree of spread; M: presence of distant metastasis) classification of Union for International Cancer control (UICC) and the summary of selected cohort was published by Zhi *et al.* [5]. The current study was approved by the Institutional Review Board of University of Medical Sciences in Poznan, and informed consents were obtained from all patients.

#### Exclusion criteria

Following the study protocol, patients with local recurrences, second primary tumor and HPV positive were excluded from experimental groups. Patients with a previous history of chemo- or radiotherapy were also excluded.

#### RT² Profiler™ PCR Array Human Cancer PathwayFinderTM

Total RNA was extracted from cancer and surrounding healthy tissues from five randomly selected patients using TRIzol reagent (Life Technologies, Grand Island, NY), and the First Strand cDNA synthesis was achieved using the RT^2^ First Strand Kit (SABiosciences, Valencia, CA) following the manufacturer's instructions. The RT^2^ Profiler^TM^ PCR ArrayHuman Cancer PathwayFinder^TM^ (PAHS-033Z, SABiosciences) was applied on the 7900HT fast real-time PCR system (Life Technologies). Each 96-well RT^2^ Profiler^TM^ PCR Array Human Cancer PathwayFinder^TM^ contains 84 genes related to human cancers, five wells for different housekeeping genes, a genomic DNA contamination control, three replicate reverse transcription controls, and three replicate positive PCR controls. Data analyses were performed using the web-based analysis software (http://pcrdataanalysis.sabiosciences.com/pcr/arrayanalysis.php).

#### Quantitative RT-PCR for validation

Total RNAs from cancer and healthy tissues from all 41 patients included in this study were extracted using TRIzol reagent (Life Technologies), and subjected to reverse transcription using an iScript^TM^cDNA synthesis kit (170-8891, Bio-Rad, Hercules, CA). Quantitative PCR was performed on the ABI7900HT fast real-time PCR system using FastSYBR Green master mix with Rox (4385612, Life Technologies). Twenty-one genes screened out by RT² Profiler™ PCR arrays were evaluated by quantitative RT-PCR. The primers for BCL2L11, CA9, ETS2, PGF, OCLN, CDH2, LDHA, SNAI2, VEGFC, SERPINB2, MKI67, PPP1R15A, and SLC2A1 were purchased from SABiosciences; Supplemental Table S2 lists the other primer sequences for the rest of the genes. Results were analyzed as described previously [27].

#### Statistical analyses

In order to assess the significance of the differences in gene expression levels between the normal and tumor tissue, we initially performed a standard paired t-test for each gene. Next we explored the impact of covariates on both survival and recurrence using Cox proportional-hazards (CPH) model implemented as the ‘coxph’ routine in the ‘survival’ package in R [Survival Analysis with Interval-Censored Data: A Practical Approach with R, SAS and WinBUGS (Chapman & Hall/CRC Interdisciplinary Statistics, 2014) Arnost Komarek, Kris Bogaerts, Emmanuel Lesaffre]. After identifying the important covariates to be considered in the survival and recurrence analyses, next we examined the association between the expression levels of each gene and survival and recurrence in a CPH model along with these covariates (which included age and whether the tumor was in the larynx). We pursued these analyses taking into account the following parameters: the normal tissue gene expression values, the tumor tissue gene expression analyses, and the difference between the normal and tumor tissues as predictors of survival and recurrence. Finally, we performed multivariate analyses of survival and recurrence including all the genes in a single CPH model considering age and the fact that whether the tumor was in the larynx as covariates. The analysis was repeated with normal tissue expression values, tumor tissue expression values, and the difference between normal and tumor tissue values. In the multivariate analyses involving the normal tissue and the difference between normal and tumor tissue expression values for recurrence, the models that included all the genes did not converge; hence, we excluded the genes with the smallest effect based on the univariate analyses until the models converged. This led to the exclusion of three genes from each analysis.

## SUPPLEMENTARY MATERIAL, FIGURES AND TABLES


