# Supplementary material for: Gene expression analysis of head and neck squamous cell carcinoma survival and recurrence
Source: Oncotarget. 2014 Nov 16;6(1):547–55. doi: 10.18632/oncotarget.2772 (PMC4381614; doi:10.18632/oncotarget.2772)
Supplement: Supplementary file 1 [file oncotarget-06-547-s001.pdf]

Gene expression analysis of head and neck squamous cell carcinoma survival and recurrence

Supplementary Material

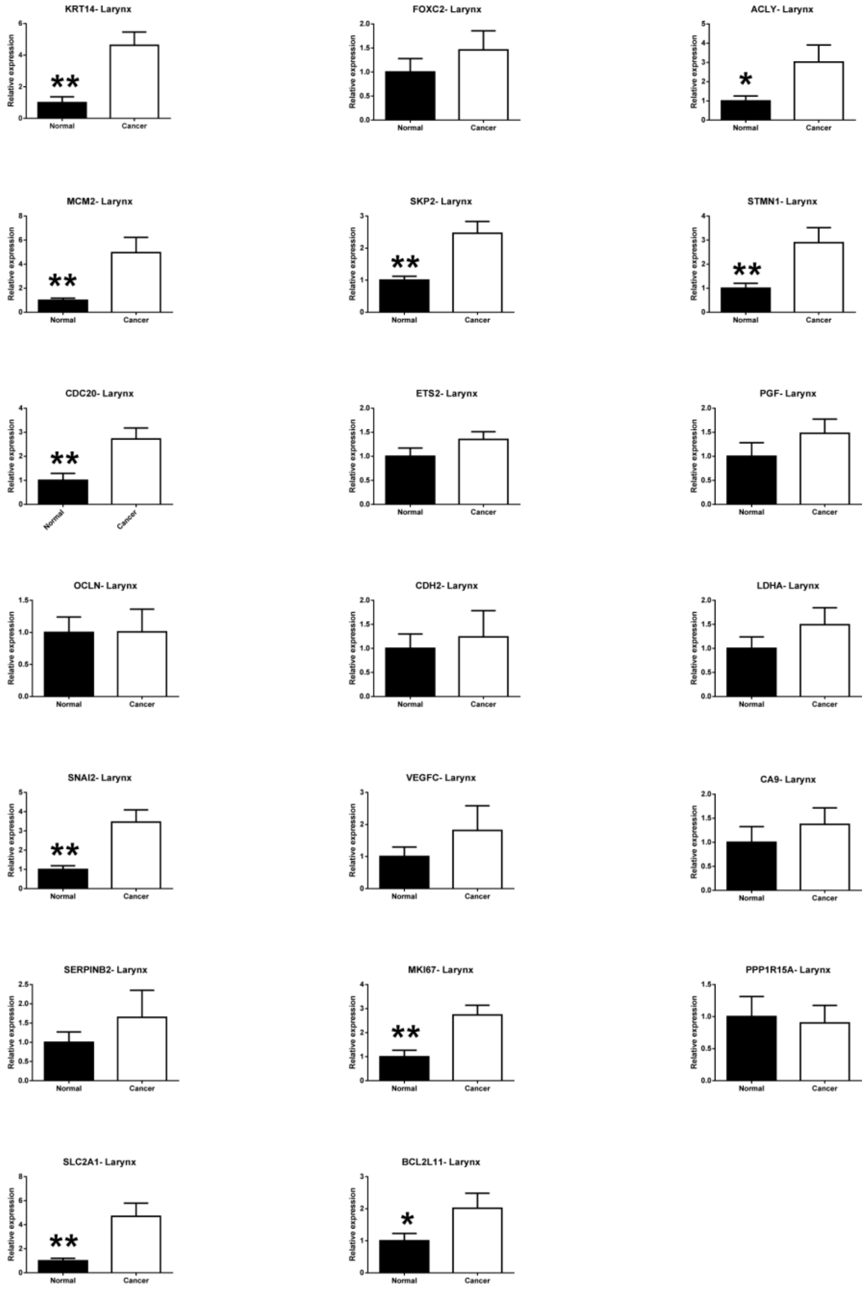

Figure S1: Gene expression between tumor and normal tissues in the larynx. The genes identified in the larynx by PCR array were investigated, 11 genes showing significant changes (*$p < 0.05$; **$p < 0.01$, *t*-test).

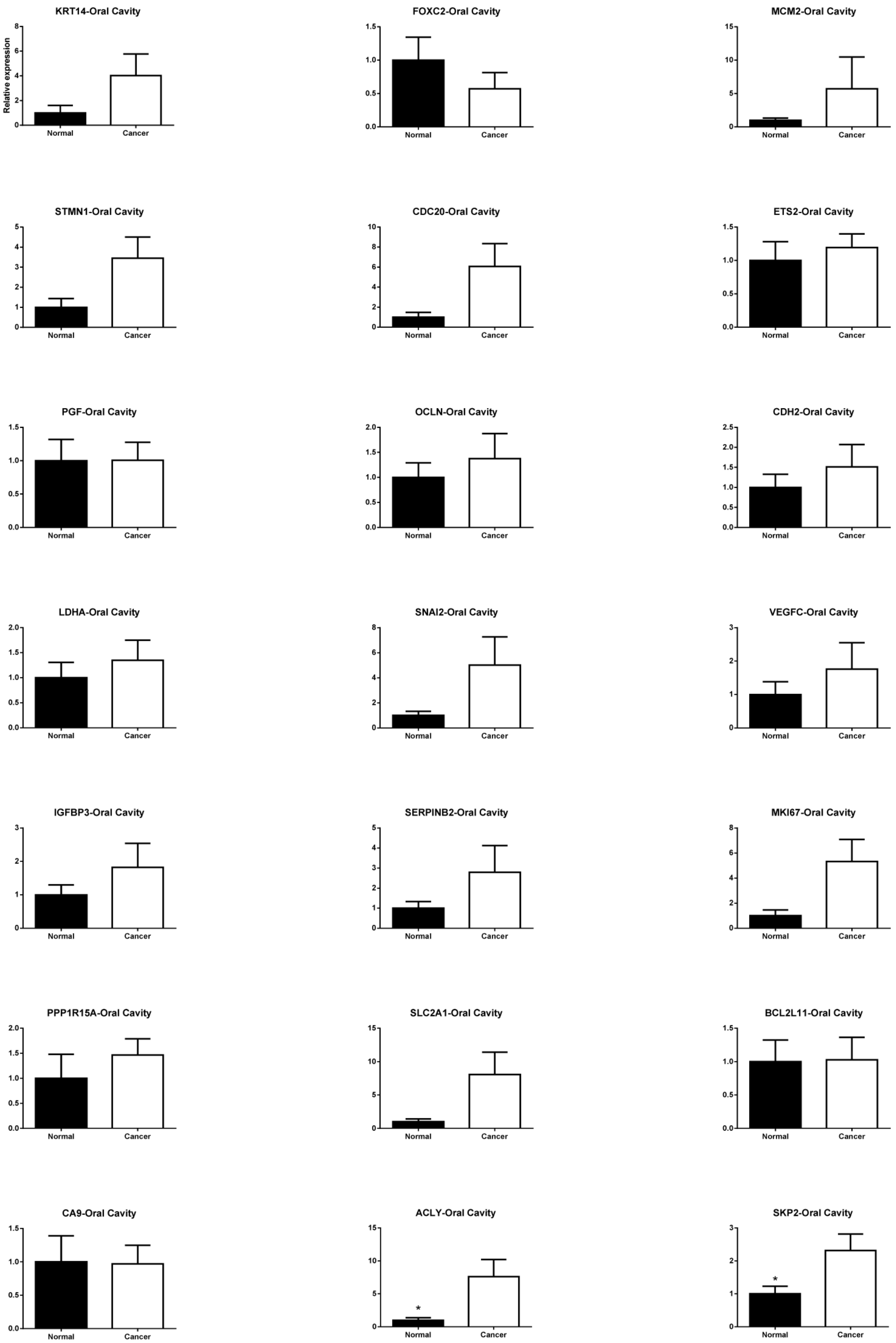

Figure S2: Gene expression between tumor and normal tissues in the oral cavity. The genes identified in the oral cavity by PCR array were investigated, two genes showing significant changes (*$p < 0.05$; *t*-test).

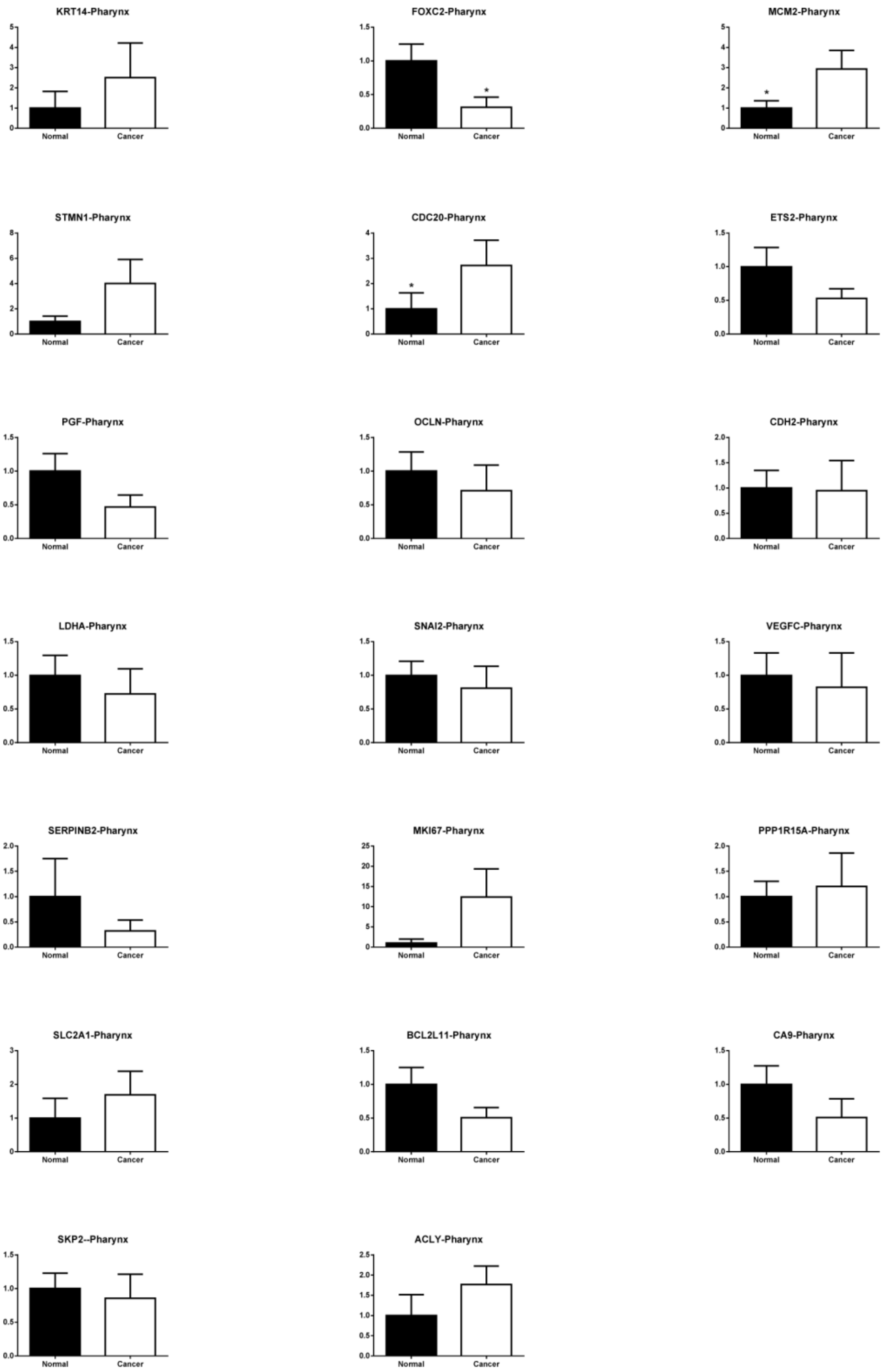

Figure S3: Gene expression between tumor and normal tissues in the pharynx. The genes identified in the pharynx by PCR array were investigated, three genes showing significant changes (*$p < 0.05$; *t*-test).

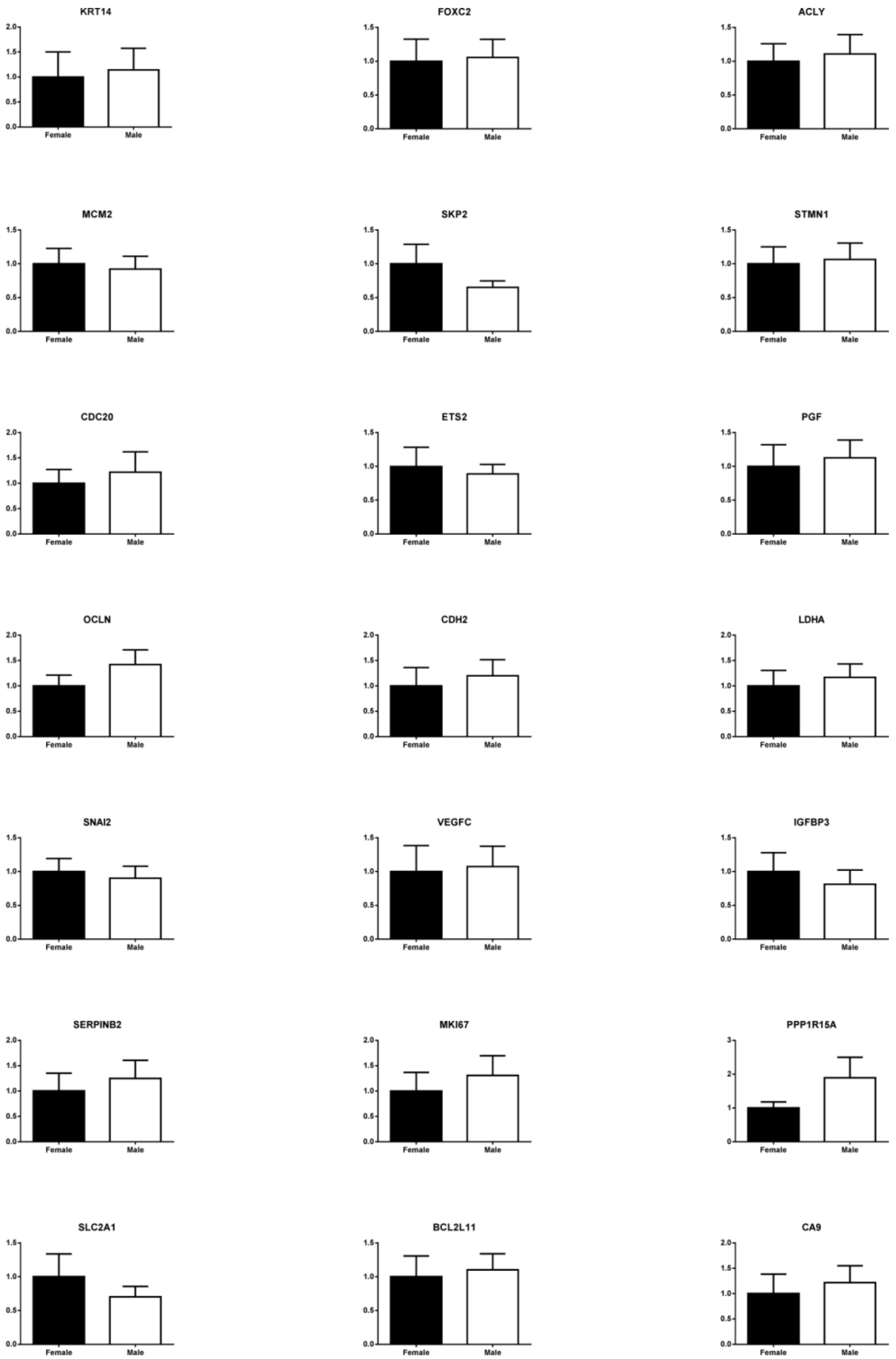

Figure S4: Differences of gene expression in normal tissues between men and women. The screened out genes were compared between men and women in normal tissue, and the results showed no obvious difference in different gender. The values in female were set as "1".

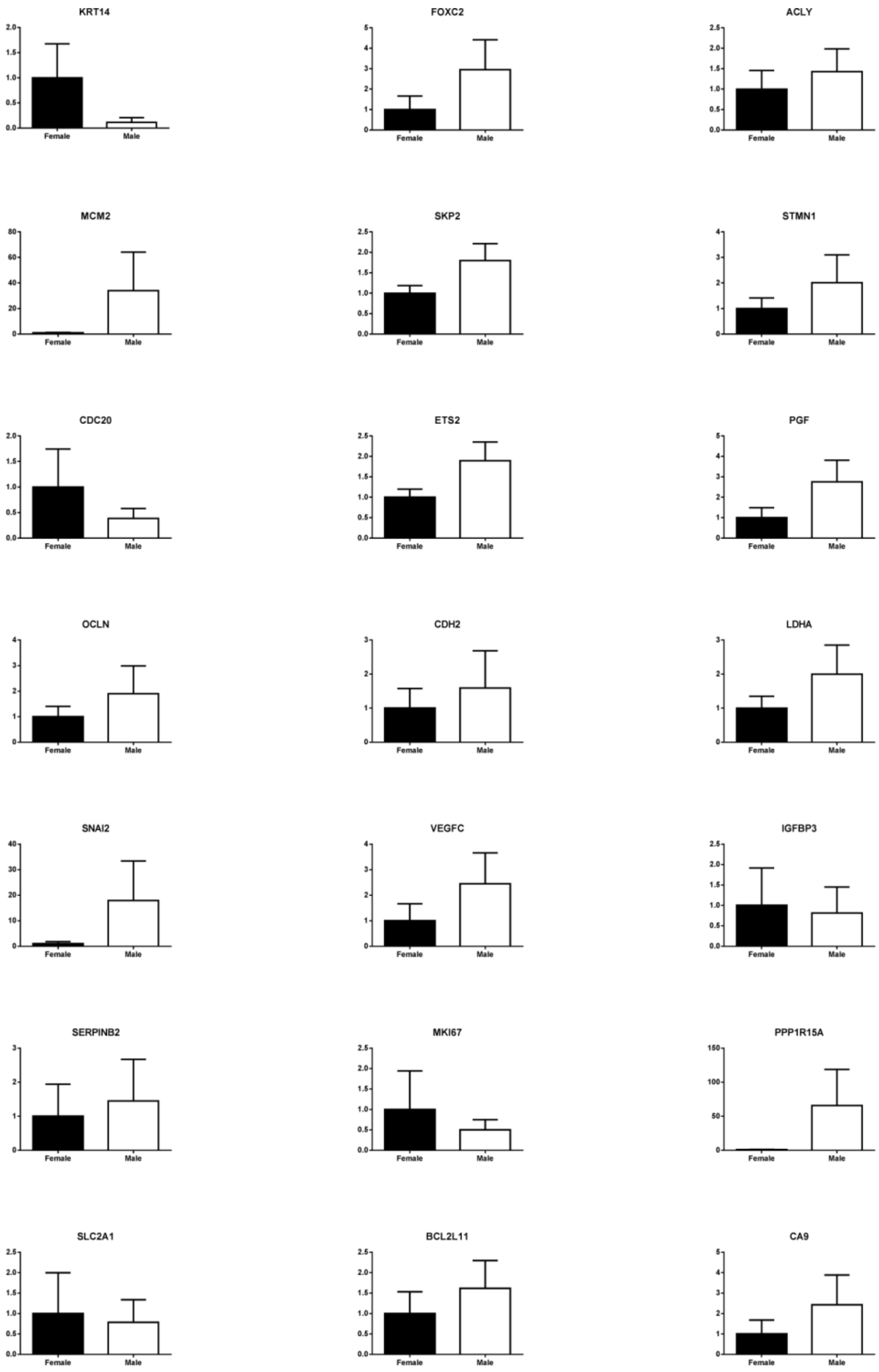

Figure S5. Differences of gene expression between men and women for tumor tissues, normalized against normal tissues. The gene expression levels were compared between men and women after the results in tumor tissue were normalized against normal tissue, and the results showed no significant differences in different gender.

A

Survival Analysis For Death					
Factor	coef	exp(coef)	se(coef)	z	Pr(>|z|)
larynx	-1.98664	0.13716	0.7713	-2.576	0.01
mthroat	-1.12549	0.32449	1.06828	-1.054	0.292
nvar	0.4987	1.64658	0.7812	0.638	0.523
tvar	0.35354	1.42411	0.3471	1.019	0.308
grade	1.00378	2.72857	0.61147	1.642	0.101
male	0.43432	1.54391	0.70443	0.617	0.538
age	0.02077	1.02099	0.03732	0.556	0.578

B

Survival Analysis For Recurrence					
Factor	coef	exp(coef)	se(coef)	z	Pr(>|z|)
larynx	-1.66311	0.18955	0.85947	-1.935	0.05
mthroat	-0.43953	0.64434	1.08338	-0.406	0.68496
nvar	-0.59119	0.55367	0.72398	-0.817	0.41417
tvar	0.58595	1.7967	0.42489	1.379	0.16788
grade	-0.21864	0.80361	0.90444	-0.242	0.80898
male	-0.28175	0.75446	0.77136	-0.365	0.71491
age	-0.12741	0.88038	0.04187	-3.043	0.00234

Table S1

**Genes**	**Sequences (5’ to 3’)**
RPLPO	Forward:<br>Reverse:
KRT14	Forward: CGC AGT CAT CCA GAG ATG TGA<br>Reverse: TCC AGT GGG ATC TGT GTC CA
FOXC2	Forward: GGC GAG CCG TCT CGG AAG<br>Reverse: CGG TAG TAA TTC TGC TCG CTC A
ACLY	Forward: GGA CTT CGG CAG AGG TAG AG<br>Reverse: TTG CCC GTC TGC TCT GAA AT
MCM2	Forward: ATC TAC GCC AAG GAG AGG GT<br>Reverse: GCT GCC TGT CGC CAT AGA TT
SKP2	Forward: TGC TAA GCA GCT GTT CCA GAC T<br>Reverse: AGC TGG GTG ATG GTC TCT GA
STMN1	Forward: CTG ATT CTC AGC CCT CGG TC<br>Reverse: AGC TTC ATG GGA CTT GCG TC
CDC20	Forward: AAT GCG CCA GAG GGT TAT CA<br>Reverse: CGG CCA GTA CAT TCC CAG AA

Table S2
